# Psychometric evaluation of an adult post-COVID-19 symptom tool: a development and validation study

**DOI:** 10.1038/s41598-024-51287-1

**Published:** 2024-01-05

**Authors:** Po-Yuan Kuo, Ping-Ho Chen, Shu-Feng Tsai, Wan-Ling Lin, Chia-Tai Hung, Sheng-Miauh Huang

**Affiliations:** 1 Taipei Medical University Hospital, Taipei, Taiwan; 2 Department of Nursing, MacKay Medical College, No. 46, Section 3, Zhongzheng Road, Sanzhi District, New Taipei City, 252 Taiwan

**Keywords:** Diseases, Health care, Medical research, Signs and symptoms

## Abstract

The objective of this study was aimed to develop and validate an instrument for post-COVID-19 symptoms in adults. Data were collected from adults with a previous COVID-19 diagnosis in Taiwan. We developed the initial instrument through systematic review and expert feedback. Its validity was tested using exploratory factor analysis (EFA), confirmatory factor analysis (CFA), and criterion-related validity, while its reliability was tested using Cronbach’s alpha. In total, 310 adults participated in this study. Examination of the EFA clearly classified a five-factor model with 24 items (Kaiser–Meyer–Olkin = 0.903; Bartlett’s test of sphericity: X^2^ = 5242.956, df = 276, p < 0.01). The goodness of fit indices of the CFA were as follows: chi-square = 635.172 (p < 0.01), normed chi-square = 2.669, standardized root mean square residual = 0.077, root mean square error of approximation = 0.073, comparative fit index = 0.922, and Tuker and Lewis index = 0.910. The value of Cronbach’s alpha coefficient for the total items was 0.941, and the values for the subscales ranged from 0.813 to 0.924. The instrument exhibited acceptable psychometric properties, proving it to be a valuable tool for evaluating post-COVID-19 symptoms in patients at hospitals.

## Introduction

Clinical presentation and outcomes in patients with coronavirus disease-2019 (COVID-19) range from asymptomatic condition to death^1^. A study from a non-hospitalized cohort showed that the prevalence rate of post-acute sequelae of COVID-19 at 30-days post-infection was 68.7%^2^. Some individuals might experience symptoms lasting 12 weeks and longer^3,4^. These persistent symptoms are known collectively as post-acute sequelae of COVID-19, post-acute COVID-19, post-COVID-19 syndrome, post-COVID-19 condition, or long COVID^5–7^. The Office for National Statistics in the UK showed that 11.7% of COVID-19 study participants would describe themselves as experiencing post-COVID-19 symptoms by 12 weeks after infection (based on self-classification)^8^. The study participants with symptoms in the acute phase had a higher percentage of self-reported symptoms 12 weeks after infection (17.7% vs. 11.7%). Such a prolonged suite of signs and symptoms may interfere with daily life and the ability to undergo routine health care^9,10^. Several scholars have reviewed existing literature on post-COVID symptoms to produce a summary of the current knowledge of this syndrome and have suggested that multidisciplinary care, involving the long-term monitoring of symptoms to identify potential complications, physical rehabilitation, mental health, and social services support, should be provided^5,11,12^.

To track symptom changes in patients with COVID-19 effectively, some countries have applied specific instruments. Research participants in the Office for National Statistics in the UK survey were asked whether they had experienced any of the 12 symptoms (fever, headache, muscle ache, weakness/tiredness, nausea/vomiting, abdominal pain, diarrhea, sore throat, cough, shortness of breath, loss of taste, and loss of smell) in the past 7 days^8,9^. The research respondents from the Understanding America Study—COVID-19 Survey were asked to report whether they had experienced the following symptoms in the past 7 days: fever or chills, runny or stuffy nose, chest congestion, cough, sore throat, sneezing, muscle or body aches, headaches, fatigue or tiredness, shortness of breath, abdominal discomfort, vomiting, hair-loss, dry skin, body temperature higher than 100.4 F or 38.0 °C, diarrhea, lost sense of smell, and skin rash^13^. Only a few studies have shown the validity and reliability of these survey questionnaires. For example, Hughes et al.^14^ applied Rasch analysis to develop a symptom burden questionnaire for long-term COVID. The questionnaire included 17 scales, with 131 items for clinical assessment, which may make it difficult to implement. In a study by Bahmer et al.^15^ the post-COVID syndrome scale, with 12 clinical symptom complexes developed by k-means clustering and ordinal logistic regression analysis, was used to survey symptom severity in Germany. However, the tool only highlights the importance of physical sequelae in different organs and systems. Current evidence supports the presence of psychological symptoms, such as anxiety or depression after acute infection in people with COVID-19^4,16–20^. Based on the above, further research is required to develop user-friendly, comprehensive, and validated tools to measure symptoms changes after acute COVID-19.

Several studies have described the occurrence of the physiological and psychological symptoms of COVID-19^2,11,21^. A pooled prevalence data showed that the 10 most reported symptoms were fatigue, shortness of breath, muscle pain, joint pain, headache, cough, chest pain, altered smell, altered taste, and diarrhea^11^. Cardiopulmonary symptoms, including heart palpitations or tachycardia, are prevalent and are associated with significant disability and heightened anxiety^21,22^. Neurological ailments (e.g. vertigo, headache, sensory deficits, numbness, anosmia, ageusia, memory issues, deficits in concentration or cognition), depression, and sleep disturbance were also found at 4–12 months after infection^11,15,23,24^. Recently, it was reported that some people experienced persistent and prolonged salivary dysfunction and gastrointestinal symptoms (e.g. diarrhea, lack of appetite) after the acute illness of the infection had healed^12,25^. Almost all studies emphasize the occurrence, but not the severity, of symptoms. The development of a new post-COVID-19 instrument to measure the severity of physical and psychological symptoms could help clinical staff to observe changes in overall symptoms and serve as an indicator to guide treatment.

Against this background, we aimed to develop and validate a set of patient-reported instruments to monitor symptom severity after the acute phase of COVID-19. We defined post-COVID-19 symptoms as one or more physical and psychological symptoms that occur in individuals with a history of COVID-19 infection, typically persisting 3 months after the onset of COVID-19, which cannot be explained by other diagnoses.

## Methods

### Study design

A cross-sectional, descriptive, explorative study design was used to develop the new Post-COVID-19 Symptom Scale (PCSS), which was conceived to measure symptom severity in patients with a previous diagnosis of COVID-19. The scale was developed and validated using a two-stage process at a medical college in Taiwan, from February 2022 to September 2022. The study adhered to the tenets of the declaration of Helsinki.

### Instrument and procedure

#### Stage I: scale development (March to March 2022)

In the first stage, during scale development, items were generated and reduced. Relevant symptoms were first established via a literature review, through which we identified post-COVID-19 symptoms. Initially, a pool of 24 potential items was generated. Scales with more reference points have proven to decrease the measurement error^26^. Hence, responses to each item were based on an 11-point Likert scale^26^. A higher scale score indicated more severe symptoms. In addition, the scale was refined using ratings from five experts: we recruited five healthcare providers with expertise in ENT (Ear, Nose and Throat), infection, chest medicine, Chinese medicine, or nursing and asked them to rate the original 24 items of the new PCSS in terms of three domains: relevance, importance, and appropriateness. This rating was based on a 4-point scale; the higher the score, the more relevant, important, or appropriate the item was considered. Finally, a 24-item PCSS was generated. They then listed the reasons for revising certain items and provided specific suggestions.

#### Stage II: scale validation (June to December 2022)

To validate the scale, we recruited patients from a medical university hospital in Taiwan, who were older than 18 years, diagnosed with COVID-19 at least 3 months, and who could communicate in Chinese. Individuals with previous cognitive disorders (e.g. dementia) were excluded because they could not complete or answer the questionnaire. Based on the report of Tinsley & Tinsley^27^ regarding sample sizes (a ratio of 5 to 10 participants per item), the minimum sample size was calculated as 120. After institutional review board approval, the investigator requested a list of patients with a diagnosis of COVID-19 between March and May 2022, and their contact numbers, from the hospital. Patients diagnosed with COVID-19 in March 2022 were interviewed by telephone in July 2022 based on consideration of 3 months after COVID-19 onset. By analogy, each patient in April and May. A study consent form and questionnaire were sent after verbal consent for participation was obtained from the subjects telephonically. Informed consent was obtained from all subjects. All methods were performed in accordance with the relevant guidelines and regulations. Thirty patients with a diagnosis of COVID-19 in March 2022 were assigned to a test–retest group and were additionally asked to complete the PCSS a second time within 2 weeks of the initial survey.

After collecting data, we used exploratory factor analysis (EFA) to identify the underlying components of the PCSS items. Construct validity was assessed using a confirmatory factor analysis (CFA). Moreover, in previous studies, females under the age of 50 years were significantly more likely to report residual symptoms^3,28–30^. Accordingly, we expected that a higher PCSS would be associated with female sex or younger age. Criterion-related validity was assessed by investigating its interference in daily life, based on the definition of COVID. A six-item measurement was used to assess life interference (general activity, mood, work, relationships with others, walking, and enjoyment of life). The six questions were rated on a 0–10 scale (0 = “no interference” to 10 = “strongly interference”) and formed the criteria used in our study. A higher score indicated stronger interference. We expected that a higher PCSS score would be associated with higher scores on criterion questions.

### Data analysis

Descriptive statistics (mean, standard deviation, frequency, and percentage) were used to illustrate the sociodemographic characteristics of the sample (age, sex, education, job, marital status, and religion). The missing values are replaced with the median value of the entire feature column. For item analysis, the independent *t*-test was used to detect whether the difference between the highest (top-27) and lowest (lowest-27) groups differed statistically (p < 0.05). A critical ratio (CR) greater than 3.5 was applied to reduce the number of items and discriminate the adequacy of each item from the subject response^31^. Statistically significant items with item total correlations of less than 0.30 or more than 0.85 were also deleted to reduce the number of items^31^.

For EFA statistics, two indicators, the Kaiser–Meyer–Olkin measure of sampling adequacy (KMO) and Bartlett’s test of sphericity, were used to determine the adequacy of the data for the factor analysis. Significantly low p values (p < 0.05) on the Bartlett test of sphericity and KMO values between 0.8 and 1 indicated that sampling was adequate^32^. The main analysis method was principal component analysis with varimax or direct oblimin rotation. The final factor solution was based on the results and a scree plot, eigenvalues > 1, factor loadings > 0.4, and percentage of variance explained. For construct validity, the goodness of fit of the factor structure was assessed using CFA. CFA analyses were performed using IBM SPSS Amos 21.0 (IBM SPSS Inc., Armonk, NY, USA). CFA was performed using the robust maximum likelihood estimator method (MLR). Based on a multifaceted approach to the assessment of model fit^33–36^ and Hoyle’s recommendations^37^, chi-square (χ^2^), normed chi-square (CMIN/DF ≈ 2), the Tuker and Lewis Index (TLI; values ≥ 0.90), comparative fit index (CFI; values ≥ 0.90), the standardized root mean square residual (SRMR; values < 0.08), and the root mean square error of approximation (RMSEA; 0.05 ≤ values ≤ 0.08 indicate a good fit) are typically considered to indicate the goodness of the model fit. The reliability of the PCSS was evaluated using Cronbach’s alpha to assess the internal consistency of each factor and overall scale. A coefficient > 0.70. considered to indicate acceptable internal consistency, and coefficients greater than 0.80 were considered to indicate good internal consistency^38^.

### Ethics approval and consent to participate

This study was approved by the ethics review committee of Taipei Medical University Hospital (N202206052).

## Results

### Sample characteristics

Overall, 412 patients during the study period met our inclusion criteria. Of these, 342 agreed to participate in the survey when contacted by telephone. The main reasons for refusal were a busy work schedule (n = 37) and privacy concerns (n = 22). For 310 of these participants, completed questionnaires were collected. The recovery rate was thus 90.64%. The participants ranged in age from 18 to 87 years (43.53 ± 13.943 years), and 82.99% were female. Table 1 shows the demographic characteristics of the participants.

**Table 1:** Characteristics of the study participants (N = 310).

Characteristic	N	%
Age (years)^a^		
≤ 50	222	71.8
≥ 51	87	28.2
Sex		
Male	122	39.4
Female	188	60.6
Educational level		
Junior high school	17	5.5
Senior high school	41	13.2
University & postgraduate	252	81.3
Currently employed		
Yes	255	17.7
No	22	82.3
Marriage status		
Single	121	39.0
Married	169	54.5
Divorced or widowed	20	6.5
Religion^a^		
Yes	180	58.3
No	129	41.7

^a^missing data = 1.

### Validity

The content validity index (CVI)^39^ of the PCSS across expert scores was 0.80, 0.85, and 0.80, respectively. None of the final PCSS items was scored as irrelevant, unimportant, or inappropriate by the five experts. Table 2 shows the mean scores and standard deviations of the individual items. All items were retained according to the item-level analyses and were further analyzed using exploratory factor analysis. Only five factors were extracted based on the screen plot results (KMO = 0.903; Bartlett’s test of sphericity: X^2^ = 5242.956, df = 276, p < 0.01). Both the direct oblimin and varimax rotation data showed a five-factor solution and a clear loading pattern. The results identified five factors explained 67.00% of the total variance, with eigenvalues > 1 (Table 3). No items were removed from the scale, and the remaining 24 items were retained for further analysis. Six of the final items assessing symptoms about cardiac, pulmonary, and central nervous system comprised one factor, called “life-threatening concern” (e.g. heart palpitations or difficulty in breathing). Four items were used to assess issues that affect thinking, comprising a second factor, called “cognitive concern” (e.g. brain fog or difficulty in concentration). Eight items assessed symptoms of psychological and physical problems. They comprised the third factor, called “psychological and non-life-threatening concern” (e.g. anxiety or dry mouth). Four items assessed perceptions of musculoskeletal pain or flu-like symptoms and comprised the fourth factor, called “ache concern” (e.g. muscle pain or sore throat). Finally, two items assessed the perceived potential olfactory and taste problems. They comprised the fifth factor, called “sensory concerns” (e.g. anosmia or ageusia).

**Table 2:** Item analysis of post-COVID-19 symptom scale (N = 310).

Items/symptom		Mean	Standard deviation	Critical ratio	Correlation to total score
1	The severity of your fatigue is…	4.15	3.11	− 20.54	0.68
2	The severity of your brain fog is…	3.09	2.99	− 19.46	0.73
3	The severity of your difficulty in remembering things is…	2.89	2.84	− 17.55	0.71
4	The severity of your difficulty in concentration is…	2.82	2.77	− 21.02	0.75
5	The severity of your anosmia is…	0.65	1.75	− 6.19	0.41
6	The severity of your ageusia is…	0.59	1.63	− 5.99	0.47
7	The severity of your headache is…	2.07	2.57	− 14.65	0.70
8	The severity of your vertigo is…	1.93	2.55	− 15.41	0.73
9	The severity of your sleep disturbance is…	2.63	3.02	− 16.43	0.61
10	The severity of your tachycardia is…	1.91	2.75	− 14.35	0.65
11	The severity of your heart palpitation is…	1.77	2.59	− 12.81	0.65
12	The severity of your difficulty in breathing is…	1.76	2.50	− 12.86	0.68
13	The severity of your cough is…	2.09	2.88	− 9.10	0.47
14	The severity of your sore throat is…	1.35	2.32	− 9.42	0.58
15	The severity of your muscle pain is…	1.65	2.50	− 11.08	0.65
16	The severity of your joint pain is…	1.41	2.28	− 9.84	0.61
17	The severity of your chest pain is…	1.23	2.15	− 10.88	0.67
18	The severity of your numbness is…	0.74	1.71	− 6.73	0.43
19	The severity of your hair loss is…	1.32	2.47	− 8.28	0.41
20	The severity of your dry mouth is…	2.20	2.66	− 14.14	0.61
21	The severity of your anxiety is…	1.71	2.45	− 15.00	0.72
22	The severity of your depression is…	1.40	2.26	− 12.33	0.71
23	The severity of your diarrhea is…	0.99	1.92	− 7.33	0.43
24	The severity of your poor appetite is…	1.05	2.05	− 8.95	0.63

**Table 3:** Factor loading after varimax rotation.

Component	Rotation sums of squared loadings		
	Total	% of variance	Cumulative %
Life-threatening concern^a^	3.72	15.51	15.51
Cognitive concern^b^	3.63	15.13	30.65
Psychological and non-life-threatening concern^c^	3.43	14.31	44.95
Ache concern^d^	3.29	13.71	58.67
Sensory concern^e^	2.00	8.33	67.00

^a^item 7, 8, 10, 11, 12, 17; ^b^item 1, 2, 3, 4; ^c^item 9, 18, 19, 20, 21, 22, 23, 24; ^d^item 13, 14, 15, 16; ^e^item 5, 6

**Figure 1:**
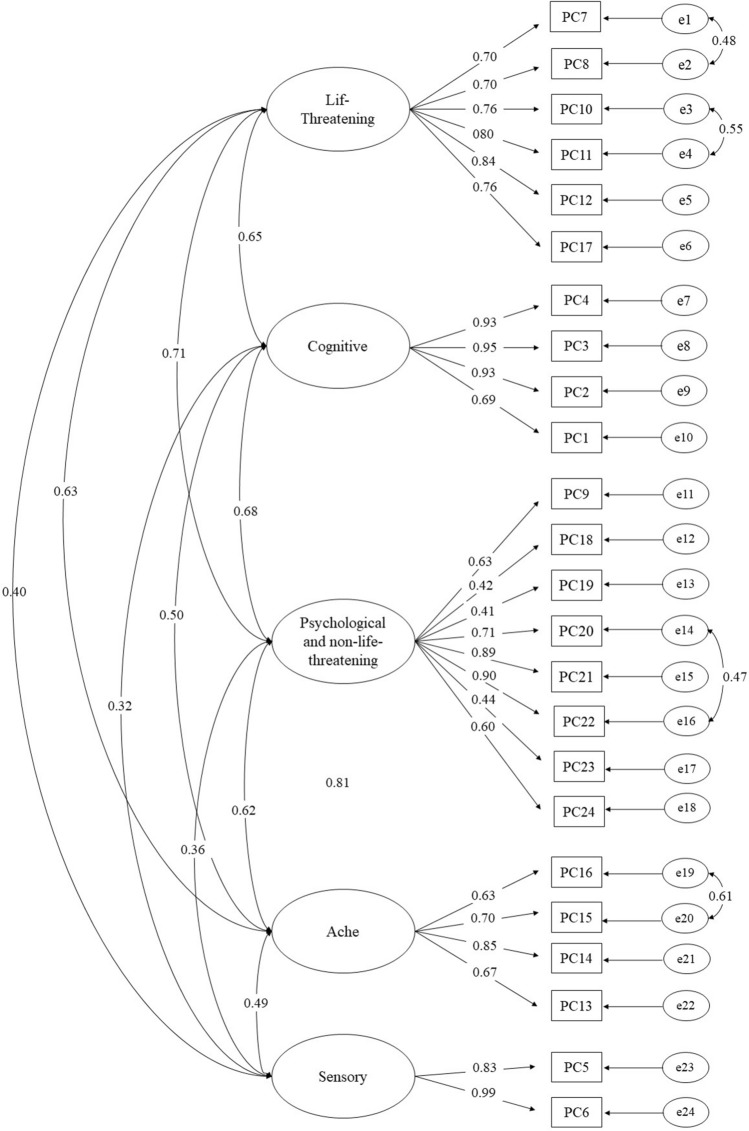
Confirmatory factor analysis of post-COVID symptom scale.

Furthermore, a CFA was conducted to verify the two models. First, a five-factor CFA was performed, without considering the modification index (Table 4, Model 1). Then, Model 1 with modification indices was used when the value of the modification index exceeded 20 (Model 2). The model fit indices are summarized in Table 4. Of the two models, Model 2 had the best model fit (Model 1: RMSEA = 0.093, SRMR = 0.075, CFI = 0.873, TLI = 0.855; Model 2: RMSEA = 0.073, SRMR = 0.077, CFI = 0.922, TLI = 0.910; Table 4). Model 2 results suggested that the five-dimensional model was the best model for cross-validation via CFA (Fig. 1).

**Table 4:** Confirmatory factor analysis fit indexes (N = 310).

	CFA index standard	Model 1	Model 2
Chi-square		893.796	635.172
DF		242	238
Normed chi-square (CMIN/DF)	≈2	3.693	2.669
RMSEA	< 0.08	0.093	0.073
SRMR	< 0.08	0.075	0.077
CFI	≥ 0.90	0.873	0.922
TLI	≥ 0.90	0.855	0.910

*CFA* confirmatory factor analysis, *CFI* comparative fit index, *DF* degree of freedom, *RMSEA* root mean square error of approximation, *SRMR* standardized root mean square residual, *TLI* Tuker–Lewis index.

Model 1: Maximum likelihood with robust standard errors, Model 2: Maximum likelihood with robust standard errors and modification indices.

Regarding known-group validity, our results showed that female patients had higher PCSS scores than male patients (49.750 ± 40.695 vs. 33.623 ± 33.485; t = -3.801; p < 0.01). Patients aged < 50 years had higher PCSS scores than those aged > 50 years (47.878 ± 40.677 vs. 32.322 ± 31.054; t = -3.613; p < 0.01). Pearson’s correlation coefficient between the total PCSS and the level of the total criterion questions was 0.566 (p < 0.01). Modest and moderate correlations between each PCSS factor and each criterion question are shown in Table 5 (r = 0.255–0.761, p < 0.05).

**Table 5:** Correlation coefficients between criterion questions and post-COVID-19 symptom scale (N = 310).

	Criterion						
	General activity	Mood	Work	Relations with others	Walking	Enjoyment of life	Sum
PCSS	0.471**	0.527**	0.546**	0.445**	0.457**	0.480**	0.566**
Life-threatening concern	0.466**	0.498**	0.569**	0.457**	0.428**	0.446**	0.553**
Cognitive concern	0.544**	0.669**	0.632**	0.547**	0.451**	0.557**	0.658**
Psychological and non-life-threatening concern	0.447**	0.440**	0.487**	0.474**	0.398**	0.524**	0.534**
Ache concern	0.275**	0.311**	0.255**	0.319**	0.287**	0.302**	0.335**
Sensory concern	0.573**	0.642**	0.661**	0.572**	0.518**	0.593**	0.688**
Sum	0.471**	0.527**	0.546**	0.445**	0.457**	0.480**	0.566**

*PCSS* post-COVID symptom scale.

**p < 0.01.

### Reliability

The reliability assessment included evaluation of internal consistency. Cronbach’s α coefficient for the 24-item PCSS overall was 0.941. Cronbach’s α coefficients ranged from 0.813 to 0.924 among the five factors. The factor-total correlations ranged from 0.497 to 0.890 (p < 0.01). The test–retest reliability coefficient for the PCSS was 0.54 (p < 0.01).

## Discussion

Accurate measurement of symptom-related changes is key in psychotherapy research and in the investigation of treatment efficacy^40^. We here described a newly developed scale that is a generic instrument designed to measure long-term COVID, to assess both physical and psychological symptoms. The strength of this study is that the initial items were developed using literature review and interviews with clinical professionals in Taiwan. Our tool measures each symptom on a scale of 0 to 11, allowing for the long-term tracking of symptom changes and for providing feedback on treatment. Compared with previous post-COVID-19 symptom assessment instruments^14,15^, we performed a validation study through exploratory and confirmatory factor analysis, known-group validity, and criterion validity based on our theoretical framework. Previous studies have shown that women under the age of 50 years were significantly more likely to report residual symptoms^3,28–30^. This is consistent with our results. These findings showed that all five factors are representative and, hence, the newly designed PCSS has good construct and criterion validity, indicating that it can be used to evaluate long-term COVID. The findings also showed that the new PCSS demonstrated good reliability based on internal consistency. Therefore, the results of our study indicated that the new PCSS is highly reliable and valid for assessing symptoms in patients with long-term COVID.

Previous studies have reported that the main symptoms of long COVID are categorized by physiological systems or body parts, such as the neurological system, digestive system, or thorax symptoms^15,41^. Our 24-item PCSS comprises five factors. Compared to the above studies, three of the factors identified in our study corresponded to the categories defined in the above studies and show a hierarchical relationship between them. In terms of urgent treatment, the priority order was life-threatening, ache, and sensory concerns. The subfactor called "cognitive concern" in our study included not only central nervous system symptoms, but also fatigue. This might be associated with the virus causing multiple neurological, respiratory, or immune responses during the early stages of COVID, and is consistent with previous studies^42,43^. Our study implied that psychological and non-life-threatening concerns are reflected in the perception of disease threat and the potential association between physical and mental conditions. For example, a previous study on Asian elderly noted that depression and anxiety were associated with a number of sleep-related problems^44^. Patients with severe hair-loss experienced psychological comorbidities, such as depression and anxiety^45^. More observational studies assessing the change in symptoms are needed to examine the association between the physical and psychological impact of COVID-19 infection further. Designing and arranging therapies tailored to different timing from the onset of COVID-19 is recommended.

Our study had some limitations. All the participants were enrolled from one hospital in Taipei. We did not survey the patients at other facilities or in other countries. We recruited only 30 patients, not all participants, to establish test–retest reliability. This sampling bias might undermine the external validity of the results and may cause a selection bias. Among psychological symptoms, only anxiety and depression were measured in our study. Adding psychological dimensions of stress^19^ and peritraumatic distress^20^ to future studies may offer a more comprehensive view. Post-COVID-19 symptoms may be a linguistically and culturally sensitive measure. Whether the identified post-COVID-19 symptoms in Taiwan are consistent with those of other countries merits further study. We did not capture qualitative data from participants. Collecting data on the comprehensiveness, relevance, and user-friendliness of PCSS might provide additional insights for further refinement. In future studies, inclusion of control group without a history of COVID-19 may provide more information, such as sensitivity and specificity.

## Conclusions

This study contributed to the body of evidence on the psychometric properties of long COVID. This validated study showed that the PCSS is an appropriate tool for measuring and assessing post-COVID-19 symptoms. Valid and reliable questionnaires can accurately measure the severity of the 24 items in long COVID. Misjudgement of changes in symptoms can delay the ideal opportunity for treatment. Our results suggested that this PCSS scale should be integrated into an early assessment tool to assess the severity of each symptom effectively among patients with acute COVID. The PCSS scale is useful for developing specific and effective strategies regarding care dilemmas in the clinical environment. A complete understanding of the severity of symptoms will enlighten clinical professionals, particularly, outpatient healthcare providers. More precise and specific treatment strategies are needed to overcome serious symptoms in patients with long-term COVID in future.

## Data Availability

The data that support the findings of this study are available from the corresponding author upon reasonable request.

## Abbreviations

CFA: Confirmatory factor analysis
COVID-19: Coronavirus-19
CFI: Comparative fit index
CR: Critical ratio
DF: Degree of freedom
EFA: Exploratory factor analysis
KMO: Kaiser–Meyer–Olkin measure of sampling adequacy
RMSEA: Root mean square error of approximation
MLR: Maximum likelihood estimator method
PCSS: Post-COVID-19 symptom scale
SRMR: Standardized root mean square residual
TLI: Tuker and Lewis index

## References

[1] Oran DP, Topol EJ (2020). Prevalence of asymptomatic SARS-CoV-2 Infection. Ann. Intern. Med..

[2] Bell ML (2021). Post-acute sequelae of COVID-19 in a non-hospitalized cohort: Results from the Arizona CoVHORT. PLoS One.

[3] Patrucco F (2022). Long-lasting consequences of coronavirus disease 19 pneumonia: A systematic review. Minerva Med..

[4] Cavicchioli M (2021). What will be the impact of the covid-19 quarantine on psychological distress? Considerations based on a systematic review of pandemic outbreaks. Healthcare.

[5] Fernández-de-Las-Peñas C, Palacios-Ceña D, Gómez-Mayordomo V, Cuadrado ML, Florencio LL (2021). Defining post-COVID symptoms (post-acute COVID, long COVID, persistent post- COVID): An integrative classification. Int. J. Environ. Res. Public Health.

[6] National Institute for Health and Care Excellence. *COVID-19 rapid guideline: Managing the long-term effects of COVID-19.* https://app.magicapp.org/#/guideline/EQpzKn/section/n3vwoL (2022).

[7] World Health Organization. *A clinical case definition of post-COVID-19 condition by a Delphi consensus.* https://www.who.int/publications/i/item/WHO-2019-nCoV-Post_COVID-19_condition-Clinical_case_definition-2021.1 (2021).

[8] Office for National Statistics. *Technical article: Updated estimates of the prevalence of post-acute symptoms among people with coronavirus (COVID-19) in the UK: 26 April 2020 to 1 August 2021.* https://www.ons.gov.uk/peoplepopulationandcommunity/healthandsocialcare/conditionsanddiseases/articles/technicalarticleupdatedestimatesoftheprevalenceofpostacutesymptomsamongpeoplewithcoronaviruscovid19intheuk/26april2020to1august2021 (2021).

[9] Office for National Statistics. *Prevalence of ongoing symptoms following coronavirus (COVID-19) infection in the UK: 1 December 2022.* https://www.ons.gov.uk/peoplepopulationandcommunity/healthandsocialcare/conditionsanddiseases/bulletins/prevalenceofongoingsymptomsfollowingcoronaviruscovid19infectionintheuk/1december2022 (2022).

[10] France K, Glick M (2022). Long COVID and oral health care considerations. J. Am. Dent. Assoc..

[11] Aiyegbusi OL (2021). Symptoms, complications and management of long COVID: A review. J. R. Soc. Med..

[12] Crook H, Raza S, Nowell J, Young M, Edison P (2021). Long covid-mechanisms, risk factors, and management. BMJ.

[13] Wu Q, Ailshire JA, Crimmins EM (2022). Long COVID and symptom trajectory in a representative sample of Americans in the first year of the pandemic. Sci. Rep..

[14] Hughes SE (2022). Development and validation of the symptom burden questionnaire for long covid (SBQ-LC): Rasch analysis. BMJ.

[15] Bahmer T (2022). Severity, predictors and clinical correlates of post-COVID syndrome (PCS) in Germany: A prospective, multi-centre, population-based cohort study. EClinicalMedicine.

[16] Dubey S (2020). Psychosocial impact of COVID-19. Diabetes Metab. Syndr..

[17] Huang C (2021). 6-month consequences of COVID-19 in patients discharged from hospital: A cohort study. Lancet.

[18] Meherali S (2021). Mental health of children and adolescents amidst COVID-19 and past pandemics: A rapid systematic review. Int. J. Environ. Res. Public Health.

[19] Thiyagarajan A, James TG, Marzo RR (2022). Psychometric properties of the 21-item depression, anxiety, and stress scale (DASS-21) among Malaysians during COVID-19: A methodological study. Humanit. Soc. Sci. Commun..

[20] Marzo RR (2021). Psychological distress during pandemic Covid-19 among adult general population: Result across 13 countries. Clin. Epidemiol. Glob. Health.

[21] Bisaccia G (2021). Post-acute sequelae of COVID-19 and cardiovascular autonomic dysfunction: What do we know?. J. Cardiovasc. Dev. Dis..

[22] Raman B, Bluemke DA, Lüscher TF, Neubauer S (2022). Long COVID: Post-acute sequelae of COVID-19 with a cardiovascular focus. Eur. Heart J..

[23] Augustin M (2021). Post-COVID syndrome in non-hospitalised patients with COVID-19: A longitudinal prospective cohort study. Lancet. Reg. Health Eur..

[24] Premraj L (2022). Mid and long-term neurological and neuropsychiatric manifestations of post-COVID-19 syndrome: A meta-analysis. J. Neurol. Sci..

[25] Okada Y, Yoshimura K, Toya S, Tsuchimochi M (2021). Pathogenesis of taste impairment and salivary dysfunction in COVID-19 patients. Jpn. Dent. Sci. Rev..

[26] Saris WE, De Rooij K, Saris WE (1988). What kind of terms should be used for reference points?. Variation in Response Functions: A Source of Measurement Error in Attitude Research.

[27] Tinsley HE, Tinsley DJ (1987). Uses off factor analysis in counseling psychology research. J. Couns. Psychol..

[28] Asadi-Pooya AA (2022). Long COVID syndrome-associated brain fog. J. Med. Virol..

[29] Sigfrid L (2021). Long Covid in adults discharged from UK hospitals after Covid-19: A prospective, multicentre cohort study using the ISARIC WHO clinical characterisation protocol. Lancet Reg. Health Eur..

[30] Sykes DL (2021). Post-COVID-19 symptom burden: What is long-COVID and how should we manage it?. Lung.

[31] Kelley TL (1939). The selection of upper and lower groups for the validation of test items. J. Educ. Psychol..

[32] Fabrigar L (1999). Evaluating the use of exploratory factor analysis in psychological research. Psychol. Methods.

[33] Hu LT, Bentler PM (1998). Fit indices in covariance structure modeling: Sensitivity to underparameterized model misspecification. Psychol. Methods.

[34] Hu LT, Bentler PM (1999). Cutoff criteria for fit indexes in covariance structure analysis: Conventional criteria versus new alternatives. Struct. Equ. Model..

[35] MacCallum RC, Austin JT (2000). Applications of structural equation modeling in psychological research. Annu. Rev. Psychol..

[36] Tanaka JS, Bollen KA, Long JS (1993). Multifaceted conceptions of fit in structural equation models. Testing Structural Equation Models.

[37] Hoyle RH (1995). Structural Equation Modeling: Concepts, Issues, and Applications.

[38] Nunnally JC, Bernstein IH (1994). Psychometric Theory.

[39] Davis LL (1992). Instrument review: Getting the most from your panel of experts. Appl. Nurs. Res..

[40] Karin E, Dear BF, Heller GZ, Gandy M, Titov N (2018). Measurement of symptom change following web-based psychotherapy: Statistical characteristics and analytical methods for measuring and interpreting change. JMIR Ment. Health.

[41] Tran VT (2022). Development and validation of the long coronavirus disease (COVID) symptom and impact tools: A set of patient-reported instruments constructed from patients’ lived experience. Clin. Infect. Dis..

[42] Ceban F (2022). Fatigue and cognitive impairment in post-COVID-19 syndrome: A systematic review and meta-analysis. Brain Behav. Immun..

[43] Miskowiak KW (2021). Cognitive impairments four months after COVID-19 hospital discharge: Pattern, severity and association with illness variables. Eur. Neuropsychopharmacol..

[44] Yu J (2016). Sleep correlates of depression and anxiety in an elderly Asian population. Psychogeriatrics.

[45] Do Nascimento IJ (2020). Effect of oral minoxidil for alopecia: Systematic review. Int. J. Trichol..

